# *Stegomyia* Indices and Risk of Dengue Transmission: A Lack of Correlation

**DOI:** 10.3389/fpubh.2020.00328

**Published:** 2020-07-24

**Authors:** Triwibowo Ambar Garjito, Muhammad Choirul Hidajat, Revi Rosavika Kinansi, Riyani Setyaningsih, Yusnita Mirna Anggraeni, Mujiyanto, Wiwik Trapsilowati, Jastal, Ristiyanto, Tri Baskoro Tunggul Satoto, Laurent Gavotte, Sylvie Manguin, Roger Frutos

**Affiliations:** ^1^Institute for Vector and Reservoir Control Research and Development, National Institute of Health Research Development (NIHRD), MoH, Salatiga, Indonesia; ^2^Université de Montpellier, Montpellier, France; ^3^HydroSciences Montpellier (HSM), Institut de Recherche pour le Développement (IRD), CNRS, Université de Montpellier, Montpellier, France; ^4^Health Research and Development Unit Banjarnegara, National Institute of Health Research Development (NIHRD), MoH, Banjarnegara, Indonesia; ^5^Department of Parasitology, Faculty of Medicine, Public Health and Nursing, Gadjah Mada University, Yogyakarta, Indonesia; ^6^ISEM, Université de Montpellier, Montpellier, France; ^7^CIRAD, Intertryp, Montpellier, France; ^8^IES, Université de Montpellier-CNRS, Montpellier, France

**Keywords:** *Stegomyia* indices, dengue, incidence, mosquito-borne disease, *Aedes aegypti*, *Aedes albopictus*

## Abstract

Dengue is present in 128 countries worldwide and is still expanding. There is currently no treatment or universally approved vaccine available. Therefore, prevention and control of mosquito vectors remain the most efficient ways of managing the risk of dengue outbreaks. The *Stegomyia* indices have been developed as quantitative indicators of the risk of dengue outbreaks. However, conflictual data are circulating about their reliability. We report in this article the first extensive study on *Stegomyia* indices, covering 78 locations of differing environmental and socio-economic conditions, climate, and population density across Indonesia, from West Sumatra to Papua. A total of 65,876 mosquito larvae and pupae were collected for the study. A correlation was found between incidence and human population density. No correlation was found between the incidence of dengue and the *Stegomyia* indices.

## Introduction

Dengue is one of the most widespread mosquito-borne arbovirus disease worldwide. Dengue viruses are present in 128 countries worldwide with major public health, social and economic consequences (1–7). Dengue is a complex disease with a wide spectrum of clinical symptoms, ranging from asymptomatic to fatal, which is often unrecognized or misdiagnosed and confused with other fever-causing tropical diseases (8). The World Health Organization (WHO) estimates that about 390 million dengue infections occur annually, with 96 million clinical manifestation and 500,000 hospitalization (9). At least 2.5% of these hospitalizations result in death and almost half of the global world population is at risk of dengue infection (9). Southeast Asia is the most impacted region and displays the highest incidence of dengue worldwide with all four dengue serotypes circulating in most countries (1, 10).

Indonesia displays the highest dengue burden in Southeast Asia (11). First described in Jakarta and Surabaya in 1968, dengue expanded in all provinces and has become a major national health priority. The incidence of dengue has increased significantly over the past 47 years from 0.05/100,000 in 1968 to 50.75/100,000 in 2015 (12, 13). Indonesia is a hyperendemic country with all four dengue virus serotypes (DENV1 to DENV4) circulating. In 2015, the dengue endemic areas included 412 districts/municipalities out of a total of 497 (82.9%). Dengue is spreading in all human dwellings from large urban areas to small rural villages (11–15).

Dengue viruses (DENV) are mainly transmitted to humans by two species of *Aedes* mosquitoes, i.e., *Aedes aegypti* and *Aedes albopictus. Ae. aegypti* is the main dengue vector, highly anthropophilic, and well-adapted to urban life. It feeds mostly at daytime with a multiple host blood meal-seeking behavior, but can also bite at night depending on light conditions. *Ae. aegypti* breeds in a variety of artificial habitats with clear stagnant water (16). The secondary vector, *Ae. albopictus*, also known as Tiger mosquito, bites at daytime too but hosts also include animals such as amphibians, reptiles, birds and mammals. *Ae. albopictus* breeds in a wide variety of artificial and natural habitats such as tires, bamboo stumps, tree holes, etc. (17). In Indonesia, large-scale migrations from rural to urban areas over the past three decades have created slum settlements with inadequate water and sanitation facilities and poor waste management, leading to the emergence of many new breeding sites for both *Ae. aegypti* and *Ae. albopictus* (13, 14). The Indonesian climate with favorable tropical rainfall, temperature and humidity also facilitates the development of additional *Aedes* breeding sites (16). This situation has strongly increased the risk of dengue transmission in suburban areas.

The risk of dengue transmission is influenced by various factors, including trade of goods and human mobility, population density, urbanization, climate, presence of invasive populations of *Aedes* vectors and pathogens, virus evolution, density of competent vectors, and ineffective vector control strategies (18, 19). While an efficient vaccine is still under research, entomological surveillance and vector control remain the only ways to prevent and control dengue transmission (19–21). Therefore, WHO recommends a routine vector surveillance to provide a quantifiable measurement of dengue vector fluctuations and their geographical distribution for assessing the risk of outbreaks and to determine vector control interventions (2, 22). These indicators have been based on the traditional *Stegomyia* indices (HI, House Index; CI, Container Index; BI, Breteau Index) (23) to which a national Free Larva Index (FLI) was added in Indonesia. These larval and pupal indices remain the most used parameters to measure vector infestation since the capture of adult mosquitoes is labor-intensive and requires access to private premises (19, 24).

Initially, the *Stegomyia* indices were proposed to prevent and predict the risk of yellow fever transmission and critical thresholds have never been determined for dengue transmission (22, 25). A House Index (HI) threshold of 1% or less, or a Breteau Index (BI) threshold of five or less have been considered to prevent dengue transmission because of similarities in the epidemiology of dengue and yellow fever viruses (18, 26, 27). Furthermore, the Pan American Health Organization (PAHO) has divided the risk factors for dengue transmission into three levels: low (HI<0.1%), medium (0.1%<HI<5%), and high (HI>5%) (28). However, the reliability and sensitivity of the *Stegomyia* indices have been questioned (2, 19, 25, 29–31).

Until now, although several studies have been published on the reliability of the *Stegomyia* indices, no comprehensive analyses have yet been conducted. Articles were either reviews covering a broad range of regions and cases or technical articles providing quantitative data but limited to specific areas (2, 19, 25, 27, 28, 32–46). We therefore developed this study to analyze the relationship between *Stegomyia* indices and actual dengue situations over a very large zone covering 78 sampling sites throughout Indonesia from Sumatra to Papua corresponding to different locations (urban/rural) and ecosystems (coastal/non-coastal). We report here a complete analysis on the two main vectors, *Ae. aegypti* and *Ae. albopictus*.

## Materials and Methods

### Study Area

**Figure 1:**
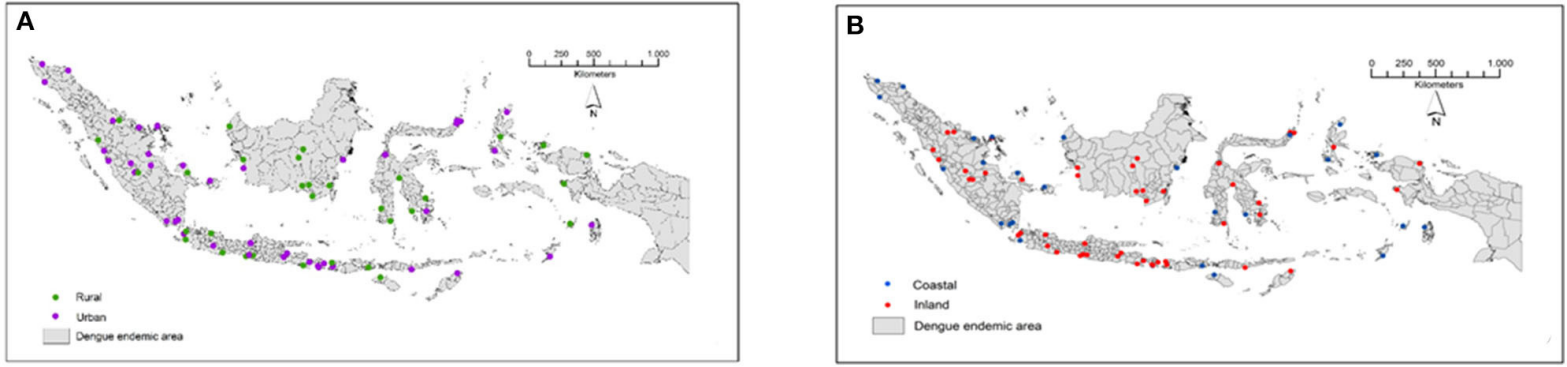
Map of the sampling sites in Indonesia. **(A)** Locations of urban and rural sampling sites. **(B)** Locations of coastal and inland sampling sites. These maps are original artworks created by the authors from a blank map background of the Republic of Indonesia displaying the district limits. This map background was provided by the Indonesian Geospatial Information Agency under agreement to use it in publication signed with IVRCRD-NIHRD.

The study was conducted in 78 locations corresponding to 78 districts/municipalities in 26 dengue-endemic provinces in Indonesia (Figure 1, Table 2). These provinces were: Aceh, Riau, Riau Islands, West Sumatra, Jambi, Bangka Belitung, Lampung, Banten, West Java, Yogyakarta, Central Java, East Java, West Kalimantan, South Kalimantan, Central Kalimantan, East Kalimantan, Southeast Sulawesi, South Sulawesi, North Sulawesi, Central Sulawesi, Bali, West Nusa Tenggara, East Nusa Tenggara, Maluku, North Maluku, and West Papua. The mosquito collection was implemented as part of the “Rikhus Vektora” project in July–August 2016 in 48 districts/cities, the WHO project SEINO 1611945 in September–October 2016 in 12 additional city locations, and finally in 18 locations in May–July 2017 as part of the Rikhus Vektora project (Figure 1).

### Study Design

The sampling plan was built using entomological data, dengue cases, socio-demographic and spatial data. Collections were undertaken at three time periods, July-August 2016 in 48 locations, September-October 2016 in 12 additional locations, and in May-July 2017 in 18 locations. These sampling periods correspond to rainy seasons in the respective locations. Each sampling periods was determined after the actual start of the rainy season and was initiated at least 1 month after the beginning of the rainy season. At least 100 households were taken at random in each sampling location to assess the presence of *Aedes* breeding sites. Three separate assessments were conducted at the same time. *Ae. aegypti* larvae and pupae, *Ae. albopictus* larvae and pupae, and *Ae. aegypti* + *Ae. albopictus* larvae and pupae were separately recorded in each sampling location. The *Stegomyia* indices were calculated for each sampling location for the three categories using the following formulas (23, 47, 48):

Container Index (CI): number of infected containers × 100/total number of containers

House Index (HI): number of infected houses × 100/total number of houses

Breteau Index (BI): number of positive containers/number of houses explored × 100

These indices were completed by a legal Indonesian index, the Free Larva Index (FLI) calculated according to the following formula:

FLI: number of houses without larva × 100/total number houses

The Free Larva Index (FLI) is the reverse of the House Index (HI) making these two indices strongly negatively correlated.

### Entomological Data Collection

Artificial and natural water-holding containers, which were potential *Aedes* breeding sites, were sampled using standardized sampling methods (23, 47–49). All pupae and larvae from positive containers were collected in separate small ziplock plastic bags. Afterwards, all samples were transported to field laboratories and counted. Due to difficulties to identify species at the larval and pupal stages, all larvae and pupae from each container were transferred to separate individual adult cages. Collected *Aedes* larvae and pupae were placed in rearing jars filled with 150 mL of freshwater and were covered with fine gauze. All larvae were fed with fish food (TetraBits, Germany). Larvae and pupae were reared until the emergence of adults for species identification.

### Sociodemographic Data Collection

The incidence, number of new dengue cases per total population for the time of the study, was considered for each community health center. Sampling locations were discriminated according to their status; i.e., urban or rural, as defined by the Ministry of Health, Republic of Indonesia, and according to the ecosystem, i.e., coastal or inland. Urban areas were defined as areas without major agricultural activity and displaying concentrations of centralized government services, social services, and economic activities. Rural areas were defined as areas having major agricultural activity, including the management of natural resources and displaying local government services, social services, and economic activities. The official discrimination between urban and rural areas is based on facilities, services, and equipment offered and not on a population density threshold. Coastal areas were terrestrial environments under marine influence whereas inland areas were far enough from the seashore to no longer be under marine influence. The number of dengue cases was taken from the national health data profile for district/city level in the time of study. The density of population (Table 1) in the zone of action of the health centers at the time of study were taken from the centralized database of health centers from the Ministry of Health, Republic of Indonesia.

**Table 1:** Population density in the sampling sites.

**Province**	**Village**	**Health center**	**Location**	**Ecosystem**	**Incidence**	**Population density (number of persons/km^**2**^)**
Aceh	Ujong Baroh	Johan Pahlawan	Urban	Coastal	0	1028.52
Aceh	Blok Benke	Kota Sigli	Urban	Coastal	67	2148.97
Aceh	Keude Aceh	Idi Rayeuk	Urban	Coastal	0	479.71
Riau	Selat Panjang Selatan	Alah Air	Urban	Coastal	122	669.52
Riau	Boncah Mahang	Sebangar	Urban	Inland	54	501.34
Riau	Bukit Kayu Kapur	Bukit Kayu Kapur	Rural	Inland	62	247.66
Riau Islands	Buliang	Batuaji	Urban	Inland	41	2917.02
Riau Islands	Tiban indah	Sekupang	Urban	Coastal	45	744.95
West Sumatra	Pakandangan	Enam Lingkung	Urban	Inland	100	485.43
West Sumatra	Aua Kuniang	Lembah Binuang	Rural	Inland	12	105.70
West Sumatra	Salido	Salido	Urban	Coastal	0	103.13
Jambi	Kenali Besar	Kenali Besar	Urban	Inland	91	1711.55
Jambi	Pinang Merah	Kenali Besar	Urban	Inland	91	1711.55
Jambi	Lubuk Kepayang	Air Hitam	Rural	Inland	0	24.12
Jambi	Jaya Setia	Muaro Bungo	Urban	Inland	210	1141.70
Jambi	Tungkal Harapan	Tungkal II	Urban	Coastal	142	1172.59
Bangka Belitung	Kuto Panji	Belinyu	Urban	Coastal	6	82.26
Bangka Belitung	Mangkol	Benteng	Rural	Inland	24	436.76
Bangka Belitung	Air Saga	Air Saga	Urban	Coastal	29	1033.30
Lampung	Jati Baru	Tanjung Bintang	Urban	Coastal	5	648.43
Lampung	Teluk Pandan	Hanura	Urban	Coastal	23	448.82
Lampung	Pasar Madang	Kota Agung	Urban	Coastal	60	545.46
Banten	Cipeucang	Binuangeun	Rural	Coastal	0	401.94
Banten	Cigondang	Labuan	Urban	Inland	0	3585.31
Banten	Ciomas	Padarincang	Rural	Inland	5	642.19
West Java	Tambak Dahan	Tambak Dahan	Rural	Inland	13	827.33
West Java	Mekargalih	Tarogong	Urban	Coastal	0	1630.22
West Java	Ciliang	Parigi	Rural	Inland	0	454.20
Yogyakarta	Kedungpoh	Nglipar II	Rural	Inland	152	401.93
Yogyakarta	Bugel	Panjatan II	Rural	Inland	151	727.26
Yogyakarta	Bangunharjo	Sewon II	Urban	Inland	360	1953.24
Central Java	Sendang Mulyo	Kedung Mundu	Urban	Inland	64	9272.12
Central Java	Sendang Guwo	Kedung Mundu	Urban	Inland	64	9272.12
East Java	Seneporejo	Silir Agung	Rural	Inland	35	920.06
East Java	Sumber Dawesari	Grati	Urban	Inland	24	1523.13
East Java	Jero	Tumpang	Urban	Inland	217	1101.93
West Kalimantan	Tengah	Kedondong	Urban	Inland	0	223.53
West Kalimantan	Pangkalan Buton	Sukadana	Rural	Inland	6	183.96
West Kalimantan	Twi Mentibar	Selakau	Rural	Coastal	0	90.74
South Kalimantan	Pabahanan	Pabahanan	Rural	Inland	31	101.27
South Kalimantan	Sungai Kupang	Sungai Kupang	Rural	Inland	14	834.65
South Kalimantan	Sumber Rahayu	Wanaraya	Rural	Inland	124	70.56
Central Kalimantan	Tampang Tumbang Anjir	Anjir	Rural	Inland	0	32.28
Central Kalimantan	Tumbang Masao	Tumbang Kunyi	Rural	Inland	0	2.87
Central Kalimantan	Kantan Muara	Pangkoh	Rural	Inland	0	39.71
East Kalimantan	Sepinggan Baru 31	Sepinggan Baru	Urban	Coastal	562	2699.96
East Kalimantan	Sepinggan Baru 59	Sepinggan Baru	Urban	Coastal	562	2699.96
South East Sulawesi	Bajo Indah	Soropia	Rural	Inland	0	1355.43
South East Sulawesi	Laea	Poleyang Selatan	Rural	Coastal	431	77.51
South East Sulawesi	Raha 3	Katobu	Urban	Inland	0	2245.73
South Sulawesi	Lestari	Tomoni	Rural	Inland	458	101.93
South Sulawesi	Palambarae	Bontonyeleng	Rural	Inland	72	536.27
South Sulawesi	Bawasalo	Segeri	Rural	Coastal	722	560.74
North Sulawesi	Bahu	Bahu	Urban	Inland	170	1576.64
North Sulawesi	Manembo Nembo Atas	Sagerat	Urban	Inland	35	905.92
North Sulawesi	Leilem	Sonder	Urban	Coastal	0	318.76
Central Sulawesi	Balaroa	Sangurara	Urban	Inland	200	3935.79
Central Sulawesi	Ujuna	Kamonji	Urban	Inland	191	5131.52
Bali	Kaliakah	Negara	Urban	Inland	325	518.09
Bali	Padang Kerta	Karangasem	Urban	Inland	1,087	1116.93
Bali	Buduk	Mengwi	Urban	Inland	1,036	2111.19
Bali	Sesetan	Denpasar Selatan I	Urban	Coastal	924	5265.03
Bali	Panjer	Denpasar Selatan I	Urban	Inland	924	5265.03
West Nusa Tenggara	Kramajaya	Narmada	Urban	Inland	17	817.78
West Nusa Tenggara	Pela	Monta	Rural	Coastal	0	149.72
West Nusa Tenggara	Medana	Tanjung	Rural	Inland	0	416.65
East Nusa Tenggara	Bairafu	Umanen	Urban	Inland	4	1486.56
East Nusa Tenggara	Nanganesa	Ngalupolo	Urban	Inland	0	140.72
East Nusa Tenggara	Wendewa Utara	Mamboro	Rural	Coastal	0	43.34
Maluku	Sifnana	Saumlaki	Urban	Coastal	0	262.43
Maluku	Siwalima	Siwalima	Urban	Coastal	0	173.50
Maluku	Faan	Watdek	Rural	Coastal	0	79.37
North Maluku	Labuha	Labuha	Urban	Coastal	0	143.68
North Maluku	Norweda	Weda	Rural	Inland	0	39.25
North Maluku	Nakamura	Daruba	Urban	Coastal	0	66.44
West Papua	Wagom Utara	Sekban	Rural	Inland	0	163.39
West Papua	Prafi Mulia	Prafi	Rural	Inland	6	50.90
West Papua	Warsadim	Warsadim	Rural	Coastal	0	3.55

### Data Analysis

A principal component analysis (PCA) was conducted using the incidence, the human population density and the four *Stegomyia* indices (HI, BI, CI, and FLI). The PCA analysis was performed on the totality of the 50 sampling locations where dengue cases have been reported by health centers. Three sets of analyses were performed separately for *Ae. aegypti, Ae. albopictus* and for the sum of *Ae. aegypti* and *Ae. albopictus* mosquitoes. The normality of the data distribution was assessed using the Kolmogorov-Smirnov normality test (50). Potential correlations between incidence and each index, and between incidence and average human densities were assessed using the Kendall τ (tau) coefficient test for rank correlation (51). This statistical test determines whether there is an ordinal association between two measured parameters. Under the null hypothesis of independence of the two datasets tested, the Kendall tau (τ) coefficient is expected to be equal to 0. Thus, a *p* > 0.05 indicates an acceptance of the null hypothesis and therefore an absence of correlation between the two datasets. The Kendall τ (tau) coefficient test for rank correlation was performed for all sites (78 sites), and only for sites were dengue cases have been recorded (50 sites). The influence of locations and ecosystems on incidence and mosquito densities was tested by Kruskal-Wallis test followed by a Siegel and Castellan *post-hoc* test for the datasets not displaying a normal distribution, and by ANOVA followed by a Bonferroni *post-hoc* test for datasets characterized by a normal distribution. All analyses were performed using Statistica v10.

## Results

### Sampling and Data Collection

Mosquitoes were collected in a total of 78 locations out of which 46 were classified as urban and 32 as rural (Figure 1, Table 2). A total of 65,876 mosquito larvae (including 55,389 *Ae. aegypti* and 10,487 *Ae. albopictus*), were collected in the 78 sampling sites (Table 2). With the exception of Warsadim in West Papua where only *Ae. malayanensis* was found, either *Ae. aegypti* or *Ae. albopictus* or both were found in all other sampling sites. Apart from Warsadim, only one site, did not host any *Ae. aegypti*, i.e., Bugel in the Province of Yogyakarta, whereas 26 sites were free of *Ae. albopictus*. The combination of *Ae. aegypti* and *Ae. albopictus* was found in 50 sampling sites (Table 2). Out of the 78 health centers analyzed, 28 did not display any case of dengue during the time of the study (Table 2). For the 50 locations displaying dengue cases, the incidence ranged from 4 in Bairafu (East Nusa Tenggara) to 1,087 in Padang Kerta (Bali) (Table 2).

**Table 2:** Entomological indices from *Aedes* larvae and pupae survey at 78 sampling sites in Indonesia.

**Province**	**Village**	**Health center^a^**	**Location**	**Ecosystem**	**Incidence**	***Aedes aegypti***					***Aedes albopictus***					***Aedes aegypti*** **+** ***Aedes albopictus***				
						**Number of *Ae. aegypti***	**HI**	**BI**	**CI**	**FLI**	**Number of *Ae. albopictus***	**HI**	**B I**	**CI**	**FLI**	**Number of *Ae. aegypti + Ae. albopictus***	**HI**	**BI**	**CI**	**FLI**
Aceh	Ujong Baroh	Johan Pahlawan	Urban	Coastal	0	402	37	51	20.82	63	254	22	24	9.79	78	656	50	75	30.61	50
Aceh	Blok Benke	Kota Sigli	Urban	Coastal	67	882	31	36	15.93	69	0	0	0	0	100	882	31	36	15.93	69
Aceh	Keude Aceh	Idi Rayeuk	Urban	Coastal	0	1,315	57	74	35.41	43	74	1	1	0.48	99	1,389	58	75	35.88	42
Riau	Selat Panjang Selatan	Alah Air	Urban	Coastal	122	157	18	28	6.39	82	187	32	46	10.5	68	344	49	74	16.89	51
Riau	Boncah Mahang	Sebangar	Urban	Inland	54	74	10	11	2.56	90	311	19	27	6.29	81	385	29	38	8.86	71
Riau	Bukit Kayu Kapur	Bukit Kayu Kapur	Rural	Inland	62	146	32	42	13.13	68	475	22	26	8.13	78	621	54	68	21.25	46
Riau Islands	Buliang	Batuaji	Urban	Inland	41	1,275	15	15	2	85	0	0	0	0	100	1,275	15	15	2	85
Riau Islands	Tiban indah	Sekupang	Urban	Coastal	45	750	11	11	4.49	89	0	0	0	0	100	750	11	11	4.49	89
West Sumatra	Pakandangan	Enam Lingkung	Urban	Inland	100	909	18	21	5.66	82	1,045	38	51	13.75	62	1,954	49	72	19.41	51
West Sumatra	Aua Kuniang	Lembah Binuang	Rural	Inland	12	171	2	158	1.89	98	74	8	10	6.33	92	245	10	13	9.23	90
West Sumatra	Salido	Salido	Urban	Coastal	0	2,419	34	42	18.5	66	78	3	4	1.76	97	2,497	35	46	15.42	65
Jambi	Kenali Besar	Kenali Besar	Urban	Inland	91	900	34	51	16.45	66	0	0	0	0	100	900	34	51	16.45	66
Jambi	Pinang Merah	Kenali Besar	Urban	Inland	91	275	34	51	13.18	66	0	0	0	0	100	275	34	51	13.18	66
Jambi	Lubuk Kepayang	Air Hitam	Rural	Inland	0	80	25	34	11.15	75	185	13	19	6.23	87	265	38	53	17.38	62
Jambi	Jaya Setia	Muaro Bungo	Urban	Inland	210	234	44	68	15.77	56	0	0	0	0	100	234	44	68	15.77	56
Jambi	Tungkal Harapan	Tungkal II	Urban	Coastal	142	862	90	315	41.39	10	0	0	0	0	100	862	90	315	41.39	10
Bangka Belitung	Kuto Panji	Belinyu	Urban	Coastal	6	1,270	31	36	13.23	69	212	10	11	4.04	90	1,482	37	47	17.28	63
Bangka Belitung	Mangkol	Benteng	Rural	Inland	24	1,291	33	39	11.75	67	1,357	32	42	12.65	68	2,648	59	81	24.39	41
Bangka Belitung	Air Saga	Air Saga	Urban	Coastal	29	122	30	32	10.45	70	214	14	7.52	23	86	336	42	55	17.97	58
Lampung	Jati Baru	Tanjung Bintang	Urban	Coastal	5	20	4	4	1.98	96	134	10	12	5.94	90	154	14	16	7.92	86
Lampung	Teluk Pandan	Hanura	Urban	Coastal	23	490	46	53	22.94	54	68	3	3	1.29	97	558	47	56	24.24	53
Lampung	Pasar Madang	Kota Agung	Urban	Coastal	60	619	16	16	6.75	84	272	21	21	8.86	79	891	30	37	15.61	70
Banten	Cipeucang	Binuangeun	Rural	Coastal	0	541	39	50	25.64	61	18	4	4	2.05	96	559	42	54	27.69	58
Banten	Cigondang	Labuan	Urban	Inland	0	122	47	58	23.02	53	45	3	3	1.19	97	167	48	61	24.21	52
Banten	Ciomas	Padarincang	Rural	Inland	5	80	40	50	20.41	60	0	0	0	0	100	80	40	50	20.41	60
West Java	Tambak Dahan	Tambak Dahan	Rural	Inland	13	595	18	18	8.65	82	27	2	2	0.96	98	622	20	20	9.62	80
West Java	Mekargalih	Tarogong	Urban	Coastal	0	1,041	29	35	14.34	71	0	0	0	0	100	1,041	29	35	14.34	71
West Java	Ciliang	Parigi	Rural	Inland	0	28	4	4	1.78	96	175	10	10	4.44	90	203	12	14	6.22	88
Yogyakarta	Kedungpoh	Nglipar II	Rural	Inland	152	5	5	8	2.15	95	349	36	49	13.17	64	354	41	57	15.32	59
Yogyakarta	Bugel	Panjatan II	Rural	Inland	151	0	0	0	0	100	82	23	27	9.82	77	82	23	27	9.82	77
Yogyakarta	Bangunharjo	Sewon II	Urban	Inland	360	160	26	52	14.36	74	0	0	0	0	100	160	26	52	14.36	74
Central Java	Sendang Mulyo	Kedung Mundu	Urban	Inland	64	482	18	19	7.53	82	0	0	0	0	100	482	18	19	7.53	82
Central Java	Sendang Guwo	Kedung Mundu	Urban	Inland	64	402	16	21	10.24	84	0	0	0	0	100	402	16	21	10.24	84
East Java	Seneporejo	Silir Agung	Rural	Inland	35	284	14	15	10.27	86	135	6	6	4.11	94	419	21	21	14.38	79
East Java	Sumber Dawesari	Grati	Urban	Inland	24	1,530	33	37	21.51	67	0	0	0	0	100	1,530	33	37	21.51	67
East Java	Jero	Tumpang	Urban	Inland	217	33	23	23	9.91	77	448	4	4	1.72	96	481	26	27	11.64	74
West Kalimantan	Tengah	Kedondong	Urban	Inland	0	2,212	84	158	38.35	16	4	1	1	0.24	99	2,216	85	159	38.59	15
West Kalimantan	Pangkalan Buton	Sukadana	Rural	Inland	6	229	20	25	7.69	80	260	17	21	6.46	83	489	37	46	14.15	63
West Kalimantan	Twi Mentibar	Selakau	Rural	Coastal	0	387	28	34	13.18	72	80	5	5	1.94	95	467	33	39	15.17	67
South Kalimantan	Pabahanan	Pabahanan	Rural	Inland	31	1,192	43	54	17.65	57	8	4	4	1.31	96	1,200	47	58	18.95	53
South Kalimantan	Sungai Kupang	Sungai Kupang	Rural	Inland	14	1,147	59	93	22.14	41	170	3	4	0.95	97	1,317	62	97	23.09	38
South Kalimantan	Sumber Rahayu	Wanaraya	Rural	Inland	124	3,226	51	69	20.97	49	315	6	7	2.13	94	3,541	57	76	23.71	43
Central Kalimantan	Tampang Tumbang Anjir	Anjir	Rural	Inland	0	175	15	25	4.66	85	77	36	71	13.22	64	252	51	96	17.88	49
Central Kalimantan	Tumbang Masao	Tumbang Kunyi	Rural	Inland	0	48	27	36	14.29	73	103	5	7	2.778	95	151	32	43	17.06	68
Central Kalimantan	Kantan Muara	Pangkoh	Rural	Inland	0	146	32	44	12.02	68	28	6	9	2.46	94	174	37	53	14.48	63
East Kalimantan	Sepinggan Baru 31	Sepinggan Baru	Urban	Coastal	562	900	61	104	35	39	0	0	0	0	100	900	61	104	35	39
East Kalimantan	Sepinggan Baru 59	Sepinggan Baru	Urban	Coastal	562	1,075	53	124	26	47	0	0	0	0	100	1,075	53	124	26	47
South East Sulawesi	Bajo Indah	Soropia	Rural	Inland	0	123	45	63	22.91	55	0	0	0	0	100	123	45	63	22.91	55
South East Sulawesi	Laea	Poleyang Selatan	Rural	Coastal	431	758	25	38	12.26	75	25	1	1	0.32	99	783	26	39	12.58	74
South East Sulawesi	Raha 3	Katobu	Urban	Inland	0	1,243	70	106	30.73	30	67	23	25	7.24	77	1,310	93	131	37.97	7
South Sulawesi	Lestari	Tomoni	Rural	Inland	458	103	27	30	6.61	73	53	15	18	3.96	85	156	30	48	10.57	70
South Sulawesi	Palambarae	Bontonyeleng	Rural	Inland	72	240	32	70	14.99	68	239	6	11	2.36	94	479	48	81	17.34	52
South Sulawesi	Bawasalo	Segeri	Rural	Coastal	722	281	87	141	21.33	13	138	46	51	7.72	54	419	87	192	29.05	13
North Sulawesi	Bahu	Bahu	Urban	Inland	170	407	13	13	7.1	87	0	0	0	0	100	407	13	13	7.1	87
North Sulawesi	Manembo Nembo Atas	Sagerat	Urban	Inland	35	224	23	28	10.18	77	30	25	29	10.18	75	254	44	57	20.73	56
North Sulawesi	Leilem	Sonder	Urban	Coastal	0	423	26	40	13.65	74	152	7	10	3.41	93	575	32	50	17.06	68
Central Sulawesi	Balaroa	Sangurara	Urban	Inland	200	950	32	52	10.55	68	0	0	0	0	100	950	32	52	10.55	68
Central Sulawesi	Ujuna	Kamonji	Urban	Inland	191	1,025	26	30	7.73	74	0	0	0	0	100	1,025	26	30	7.73	74
Bali	Kaliakah	Negara	Urban	Inland	325	68	12	17	6.29	88	37	6	8	2.96	94	105	19	25	9.26	81
Bali	Padang Kerta	Karangasem	Urban	Inland	1,087	37	15	18	8.05	85	44	20	22	9.32	80	81	27	41	17.37	73
Bali	Buduk	Mengwi	Urban	Inland	1,036	98	25	42	16.54	75	80	20	20	7.87	80	178	45	62	24.41	55
Bali	Sesetan	Denpasar Selatan I	Urban	Coastal	924	825	23	30	11.81	77	0	0	0	0	100	825	23	30	11.81	77
Bali	Panjer	Denpasar Selatan I	Urban	Inland	924	625	30	36	11.8	70	0	0	0	0	100	625	30	36	11.8	70
West Nusa Tenggara	Kramajaya	Narmada	Urban	Inland	17	126	9	9	5.59	91	55	2	2	1.24	98	181	11	11	6.83	89
West Nusa Tenggara	Pela	Monta	Rural	Coastal	0	534	26	29	11.79	74	0	0	0	0	100	534	26	29	11.79	74
West Nusa Tenggara	Medana	Tanjung	Rural	Inland	0	55	20	20	10.26	80	0	0	0	0	100	55	20	20	10.26	80
East Nusa Tenggara	Bairafu	Umanen	Urban	Inland	4	174	41	45	26.47	59	0	0	0	0	100	174	41	45	26.47	59
East Nusa Tenggara	Nanganesa	Ngalupolo	Urban	Inland	0	2,352	52	66	33.33	48	5	2	2	1.01	98	2,357	52	68	34.34	48
East Nusa Tenggara	Wendewa Utara	Mamboro	Rural	Coastal	0	2,882	63	88	45.59	37	10	1	1	0.52	99	2,892	64	89	46.11	36
Maluku	Sifnana	Saumlaki	Urban	Coastal	0	333	72	72	26.28	28	0	0	0	0	100	333	72	72	26.28	28
Maluku	Siwalima	Siwalima	Urban	Coastal	0	2,078	60	83	36.24	40	66	3	3	1.31	97	2,144	60	86	37.55	40
Maluku	Faan	Watdek	Rural	Coastal	0	5,650	81	157	35.84	19	1,095	18	31	7.08	82	6,745	91	188	42.92	9
North Maluku	Labuha	Labuha	Urban	Coastal	0	2,160	30	44	15.02	70	859	10	28	9.56	90	3,019	33	72	24.57	67
North Maluku	Norweda	Weda	Rural	Inland	0	140	4	4	1.92	96	52	1	1	0.48	99	192	5	5	2.4	95
North Maluku	Nakamura	Daruba	Urban	Coastal	0	19	2	2	1.05	98	188	24	28	14.66	76	207	26	30	15.71	74
West Papua	Wagom Utara	Sekban	Rural	Inland	0	583	77	187	33.33	23	28	20	22	3.92	80	611	77	209	37.25	23
West Papua	Prafi Mulia	Prafi	Rural	Inland	6	170	54	80	15.59	46	0	0	0	0	100	170	54	80	15.59	46
West Papua	Warsadim	Warsadim	Rural	Coastal	0	0^b^	0	0	0	100	0^b^	0	0	0	100	0^b^	0	0	0	100

*HI, House Index; CI, Container Index; BI, Breteau Index; FLI, Free Larva Index*.

a *Health Centers are Community Health Centers (CHC) or Puskesmas in Indonesian. They are government-mandated community health clinics providing healthcare for population on sub-district. These clinics are present in every sub-districts*.

b *All mosquitoes collected were Aedes malayanensis*.

### Data Normality

**Figure 2:**
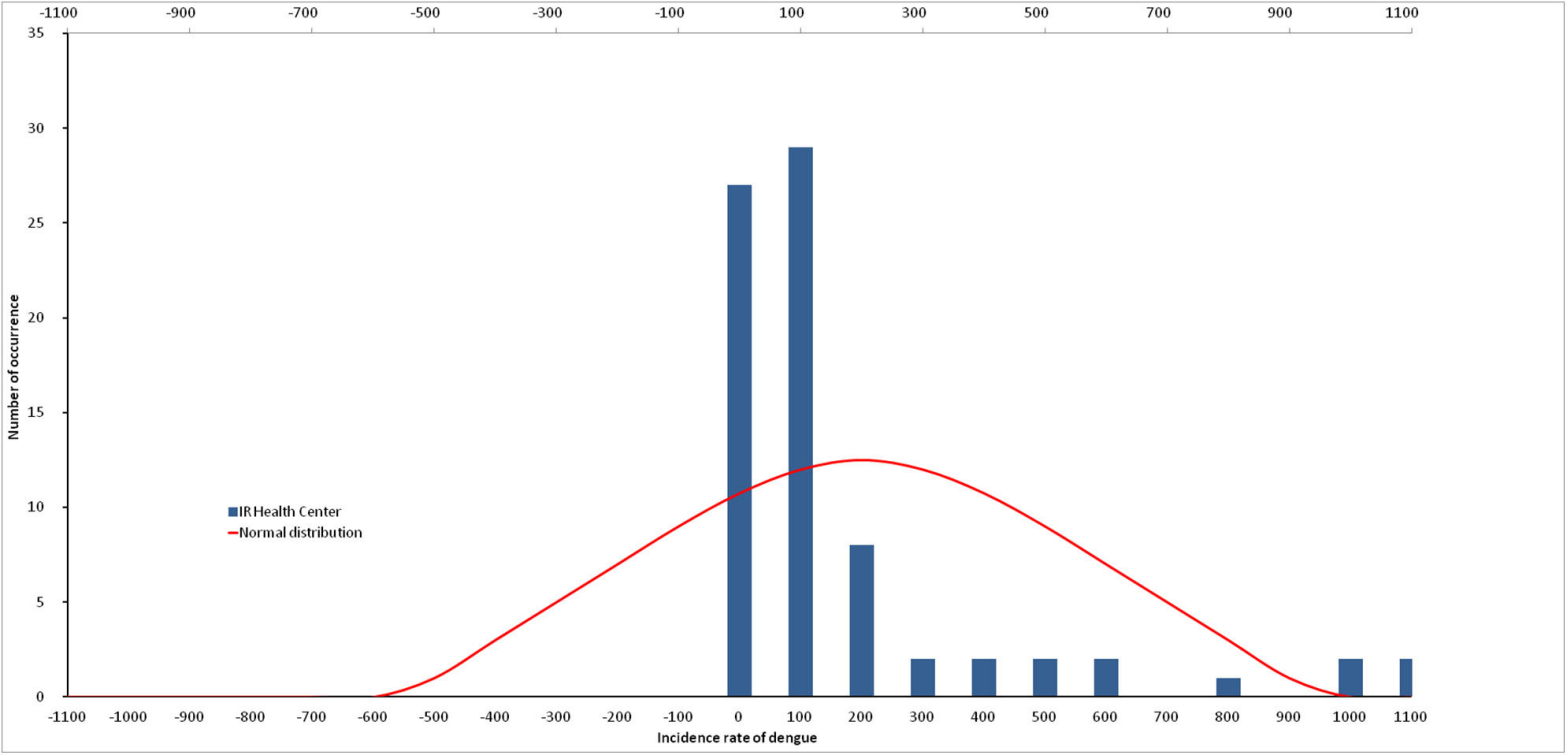
Non-normal distribution of dengue incidence.

The D-statistic from Kolmogorov-Smirnov normality test for dengue incidence indicates that the data do not follow a normal distribution (*p* = 0.002; Figure 2). Similarly, the number of mosquito larvae caught does not follow a normal distribution for *Ae. aegypti* (*p* = 0.0492), as well as for *Ae. albopictus* (*p* = 0.0023). The sum of all *Ae. aegypti* and *Ae. albopictus* larvae was the only dataset following a normal distribution (*p* = 0.0751).

### Correlation Between Dengue Infection Rates and Human Density

**Figure 3:**
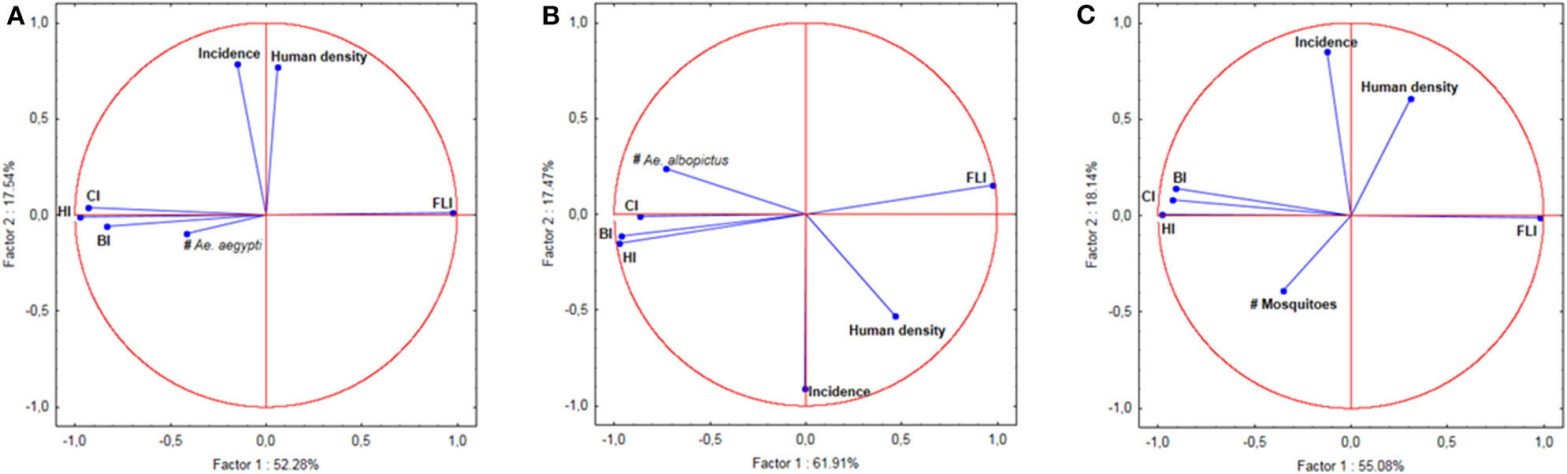
Principal Component Analysis (PCA) of indices, number of mosquitoes, human population density, and incidence of dengue. **(A)** PCA for *Ae. aegypti*. **(B)** PCA for *Ae. albopictus*. **(C)** PCA for *Ae. aegypti* and *Ae. albopictus* together.

The PCA analysis indicated a clear correlation between dengue incidence and the human population density registered for each location (Figure 3). This correlation was confirmed by the Kendall rank correlation coefficients test (τ = 0.242; *p* = 0.0125), indicating that the dengue incidence increased along with the human population density.

### Correlation Between Dengue Infection Rates and Larvae Indices

Tests on the value of the coefficient τ (Kendall rank correlation coefficients test) for the incidence of each sampling location vs. each of the indices at the same location were systematically higher than the limit *p*-value of 0.05 indicating that the test was significant. Only places clinical dengue cases have been recorded were considered in the analysis. The null hypothesis of independence of the data was therefore accepted indicating that there was no correlation between the incidences, any of the indices (CI, HI, BI and FLI) and the number of mosquitoes in all of the 50 epidemic locations analyzed (Table 3). This lack of correlation was observed for *Ae. aegypti* alone, for *Ae. albopictus* alone and for the sum of *Ae. aegypti* and *Ae. albopictus* (Table 3). The Principal Component Analysis (PCA) displayed a very high level of explanation for the datasets tested (Figure 3). For *Ae. aegypti* alone, the PCA explained 69.82% of the data spread (axis 1: 52.28% and axis 2: 17.54%) (Figure 3A). For *Ae. albopictus* alone, the PCA explained 79.38% of the data spread (axis 1: 61.91% and axis 2: 17.47%) (Figure 3B). For both species, i.e., *Ae. aegypti* and *Ae. albopictus* considered together, the level of explanation of the data spread given by the PCA analysis was 73.22% (axis 1: 55.08% and axis 2: 18.14%) (Figure 3C). For each PCA, the same observations can be made, namely: (i) a strong autocorrelation of the different indices with each other, (ii) a correlation between the indices and the total number of mosquitoes, (iii) a correlation between dengue incidence and average human density, and finally (iv) a complete lack of correlation between dengue incidence in a study site and the Stegomyia indices shown by the orthogonal position observed in all PCA analyses between indices and incidence.

**Table 3:** Tau (τ) and *p*-values obtained for incidence and entomological indices by Kendall rank correlation coefficients test.

**Species**	**House Index**	**Breteau Index**	**Container Index**	**Free Larva Index**
**ALL LOCATIONS CONSIDERED**				
*Ae. aegypti*	τ *=* −0.101	τ *=* −0.062	τ *=* −0.134	τ *=* 0.101
	*p =* 0.1926	*p =* 0.4248	*p =* 0.0821	*p =* 0.1926
*Ae. albopictus*	τ *=* −0.039	τ *=* −0.056	τ *=* −0.057	τ *=* 0.039
	*p =* 0.6107	*p =* 0.4659	*p =* 0.4633	*p =* 0.6107
*Ae. aegypti* and *Ae. albopictus*	τ *=* −0.085	τ *=* −0.039	τ *=* −0.144	τ *=* 0.085
	*p =* 0.2731	*p =* 0.6107	*p =* 0.0506	*p =* 0.2731
**LOCATIONS WITH NO DENGUE CASES EXCLUDED**				
*Ae. aegypti*	τ *=* 0.037	τ *=* 0.065	τ *=* 0.043	τ *=* −0.037
	*p =* 0.7066	*p =* 0.5034	*p =* 0.6575	*p =* 0.7066
*Ae. albopictus*	τ *=* −0.014	τ *=* −0.023	τ *=* −0.043	τ *=* 0.014
	*p =* 0.8869	*p =* 0.8184	*p =* 0.6575	*p =* 0.8869
*Ae. aegypti* and *Ae. albopictus*	τ *=* 0.043	τ *=* 0.131	τ *=* 0.016	τ *=* −0.043
	*p =* 0.6575	*p =* 0.1808	*p =* 0.8737	*p =* 0.6575

### Influence of Locations and Ecosystems

The incidence was not significantly correlated with the different environments considered: urban vs. rural (Figure 1A) and coastal vs. inland (Figure 1B) (Kruskal-Wallis: *H* = 7.72; *p* = 0.0523). Mosquito distributions were significantly different (tested by Kruskal-Wallis non-parametric statistical test) for each type of environment for both *Ae. aegypti* (*H* = 8.43; *p* = 0.038) and *Ae. albopictus* (*H* = 7.96; *p* = 0.0468). Differences (Siegel and Castellan *post-hoc* test) were marginal and only appeared between urban/inland and urban/coastal for *Ae. aegypti* (*p* = 0.037) and between rural/inland and rural/coastal for *Ae. albopictus* (*p* = 0.0404). For the combination of both species, which is the only dataset in this work following a normal distribution, the ANOVA test indicated no difference between environments (*F* = 2.045; *p* = 0.1149).

## Discussion

Following to the use of *Stegomyia* indices to predict the risk of dengue outbreaks several articles in the literature questioned their efficiency (19, 28, 45, 46). A systematic review on the application of the *Stegomyia* indices to predict dengue outbreaks was conducted (2). Out of all the articles reviewed 15 were ranked as “weak studies” and no clear conclusion could be reached (2). Out of 13 articles directly dealing with the relationship between *Stegomyia* indices and dengue infection, 4 concluded on a correlation, 4 concluded on a lack of correlation, and 5 reported inconclusive discussions (2). More recent articles published on the subject also provided various conclusions. One article concluded on the lack of correlation (45), the second concluded on a correlation (46), and the last two were inconclusive, depending on the type of analysis performed (19, 29).

The work reported here brings explanations on the diverging conclusions reached by the previous studies. The first point to consider is that all the works previously reported on this topic were focused on a single place or a limited area. No studies were performed over a very large geographic area encompassing different local climates and environmental conditions. Therefore, each study was strongly influenced by local geographic and climatic conditions but also specific urbanization and socio-economic conditions, which could have biased the data. Furthermore, these previous studies were all independent investigations with variations in sampling schemes and methodologies, making difficult a comparative analysis. Our study is based on a very large cross-section of locations of various sizes, with different urban environments throughout all of Indonesia. The geographic coverage of this work and the integration of a large set of data into a single analysis made data smoothing possible as well as elimination of variations due to specific environments or socio-economic conditions.

Data analysis in all previous studies utilized parametric statistics. However, as reported in this work, the data considered do not follow a normal, Gaussian distribution. Since parametric statistics are not well-suited for non-normal datasets, this could well-explain the contradictory conclusions previously reported. Consequently, we applied non-parametric methods to correct for bias. The dengue vectors are anthropophilic mosquitoes (52) and therefore the distribution of breeding sites is influenced by human societal aspects (53). The real drivers behind the distribution of *Aedes* breeding sites are demography, urbanization, and socio-economic level. This is supported by the correlation observed between the density of human populations and the incidence of dengue. These societal, sociological, and economical aspects do not follow a normal distribution and therefore the distribution of mosquitoes, thus the entomological indices, as well as the incidence of dengue do not either. Consequently, our application of non-parametric statistical analysis of the data, which to our knowledge was not done in any previous studies (2, 19, 25, 28, 30, 32–46), provides a very robust statistical conclusion strengthened by the size of the study and the multiplicity of sites and conditions.

We conclude that there is no correlation between the incidence of dengue and any of the *Stegomyia* indices. The very high level of explanation provided by the PCAs is a consequence of both the nature of the data studied and the absence of correlation between incidence and indices. Indeed, the first axis (abscissa on the graphs) explains the dispersion of the indices, which are necessarily correlated since they represent different elements of the mosquito population density in a study area. The second axis (ordered on the graphs) explains the dispersion of the incidence data. The lack of correlation between the two types of data is clearly represented by the orthogonality of the vectors of the various indices with respect to dengue incidence. None of the datasets influences the position of the other. Therefore, the data dispersion occurs in each set only, which considerably increases the explanation of the axes. This total lack of correlation is observed for both *Ae. aegypti* and *Ae. albopictus*, which eliminates any possibility of species-related interaction. This is also expected since the main drivers are linked to societal aspects and both species are anthropophilic (53).

The *Stegomyia* indices are not relevant descriptors for assessing the risk of dengue outbreak. They are not related to the vector competence. These indices are simply demographic descriptors. The higher the population, the higher the value of the descriptor. However, the main reason for this discrepancy is that they are targeting the wrong level of biological significance. The *Stegomyia* indices are targeting the species level, which is a good compromise between a reasonable work investment for collecting data and a systematic level accurate enough to avoid dispersion of data. Furthermore, the species is the widely recognized level of classification for the identification of living organisms. However, a species is an intellectual construction and is not biologically relevant. The relevant level of discrimination with respect to biological functions, and therefore vector competence, is the population or subspecies (54–56). A species should be regarded as a metapopulation or the combination of crossfertile genetically distinct populations displaying differing phenotypic traits (57). The vector competence of *Aedes* and other mosquitoes was shown to be related to specific populations (16, 56, 58–60) and not to the species *per se*. Targeting the species level with demographic descriptors can thus be misleading, hence the contradictory results obtained when assessing the efficiency of *Stegomyia* indices for predicting dengue outbreaks. A very high demography of a poorly vectoring population will lead to actions of prevention in the absence of risk of outbreak, whereas a low demography of a very good vectoring population would lead to a lack of action in the presence of a high risk of outbreak.

If not related to the *Stegomyia* indices, the dengue incidence is instead statistically related to the human population density. This is not really surprising since *Aedes* mosquitoes fly an average of 250 meters around their breeding site. Considering this short distance of flight, there is more chance for an infected mosquito to find a blood meal within flying distance in densely populated area than in a dispersed habitat. Other approaches than the *Stegomyia* indices, based on societal and urbanistic parameters should then be considered. The “One house/One inspector” approach recently implemented in Indonesia by the Ministry of Health is an interesting and sound alternative to the *Stegomyia* indices based on the monitoring and elimination of breeding sites at the household level (61). The philosophy of intervention developed in Indonesia is the prevention of dengue transmission through community participation. The approach implemented is the 3M approach, i.e., covering water containers (Menutup), cleaning water containers (Menguras), and burying discarded containers (Mengubur). The implementation is under the responsibility of families in each household. At least one person in each household is in charge of monitoring *Aedes* larvae in all water storage. However, to efficiently implement surveillance and risk analysis, people must be given reliable indices. It would therefore be important to communicate on the lack of reliability of the *Stegomyia* indices and to support the development of novel, more reliable, sociology-related markers, and actions taking into account the correlation between human population density and dengue incidence such as urbanism, type of housing, or socioeconomic level.

## Data Availability Statement

All datasets generated for this study are included in the article.

## Author Contributions

TG participated in all part of the work. MH, R, RS, YA, and TS contributed to the conception and design of the study, and participated to the field work. J organized the field work. R, M, and WT did the field work and built the database. LG did the statistical analyses. SM did the supervision and corrections. RF did the analyses, supervision, and writing. All authors contributed to the article and approved the submitted version.

## Conflict of Interest

The authors declare that the research was conducted in the absence of any commercial or financial relationships that could be construed as a potential conflict of interest.
